# Supplementary material for: Dynamic phase-locking states and personality in sub-acute mild traumatic brain injury: An exploratory study
Source: PLoS One. 2023 Dec 15;18(12):e0295984. doi: 10.1371/journal.pone.0295984 (PMC10723684; doi:10.1371/journal.pone.0295984)
Supplement: S1 File — (DOCX) [file pone.0295984.s001.docx]

**List of abbreviations and definitions regarding fMRI and dMRI data analyses**

AD = axial diffusivity. Measure of water diffusion parallel to the main axis (i.e., the eigenvalue for the first eigenvector of the diffusion tensor).

BIDS = brain imaging data structure. Standard for organizing, annotating, and describing neuroimaging data.

dFC = dynamic functional connectivity. Time-varying similarity/synchronization (measured with for example correlation or phase coherence) of signals of brain regions or functional subunits (i.e., networks or components).

dMRI = diffusion MRI. MRI technique for measuring diffusion of water molecules in the brain (or other tissues). Data can be used to analyze the microstructure of brain white matter tracts.

DTI = diffusion tensor imaging. A Gaussian model for water diffusion in the brain. The diffusion tensor is a 3 × 3 matrix that describes the diffusion of water in 3 orthogonal directions, or eigenvectors. The corresponding eigenvalues express the magnitude of diffusion in each direction.

fMRI = functional magnetic resonance imaging. Technique to measure the blood oxygen level dependent (BOLD) response during performance of a certain task (e.g., cognitive, motor), or rest. The BOLD signal reflects changes in oxygenated vs. deoxygenated hemoglobin in response to increases and decreases in energy demand of brain tissue.

FA = fractional anisotropy. Relative measure on scale from 0 to 1 indicating isotropic (low FA, meaning less restricted diffusion, and occurring more equally in all directions) to anisotropic (high FA, meaning diffusion occurring predominantly in one direction) movement of water molecules. The lower the FA, the more spherical the diffusion tensor becomes; the higher the FA, the more ‘cigar-like’.

ICA = independent component analysis. Linear data-driven method to extract independent sources (i.e., independent components) from a mixture of signals. When applied to fMRI data, it is possible to identify functional brain networks. These networks can be reflected by one or multiple independent components. For example, the frontoparietal network (which is part of the cognitive control domain) is often found split up into two components, left and right. Independent components consist of voxels that share similar temporal BOLD signals (i.e., these voxels have synchronized signals). Furthermore, each component has a time course, which is the signal of this component over time. These time courses can be used for functional connectivity analyses.

K-means clustering = unsupervised (i.e., without known labels, such as diagnosis or outcome) clustering algorithm that detects hidden patterns within a dataset.

LEiDA = Leading Eigenvector Dynamics. Computational technique to identify temporal patterns of functional brain connectivity. First, the phase coherence between signals of brain regions or independent components is computed at each time point. Then, the first eigenvector is extracted per time point, to obtain the dominant connectivity pattern. Lastly, these dominant connectivity patterns across time are clustered using k-means clustering to find dynamic brain states, which are recurrent patterns of brain connectivity.

MD = mean diffusivity. Average diffusion across all axes/eigenvectors of the diffusion tensor.

PCA = principal component analysis. Linear computational technique to reduce dimensions of data. It results in principal components (eigenvectors), which are orthogonal to each other. The first component explains the largest portion of variance in the original data, the last component the smallest portion.

RD = radial diffusivity. Measure of average diffusion along the second and third diffusion axis (i.e., the eigenvalues corresponding with the second and third eigenvectors of the diffusion tensor).

rs-fMRI = resting-state fMRI. During a rs-fMRI experiment the BOLD response is measured at rest (in contrast to during performance of a task), when a subject is awake, with eyes closed or focused on a fixation cross, and not thinking about anything in particular. Data can be used for (dynamic) functional connectivity analyses.
