# Supplementary figures and images for: Dynamic phase-locking states and personality in sub-acute mild traumatic brain injury: An exploratory study
Source: PLoS One. 2023 Dec 15;18(12):e0295984. doi: 10.1371/journal.pone.0295984 (PMC10723684; doi:10.1371/journal.pone.0295984)

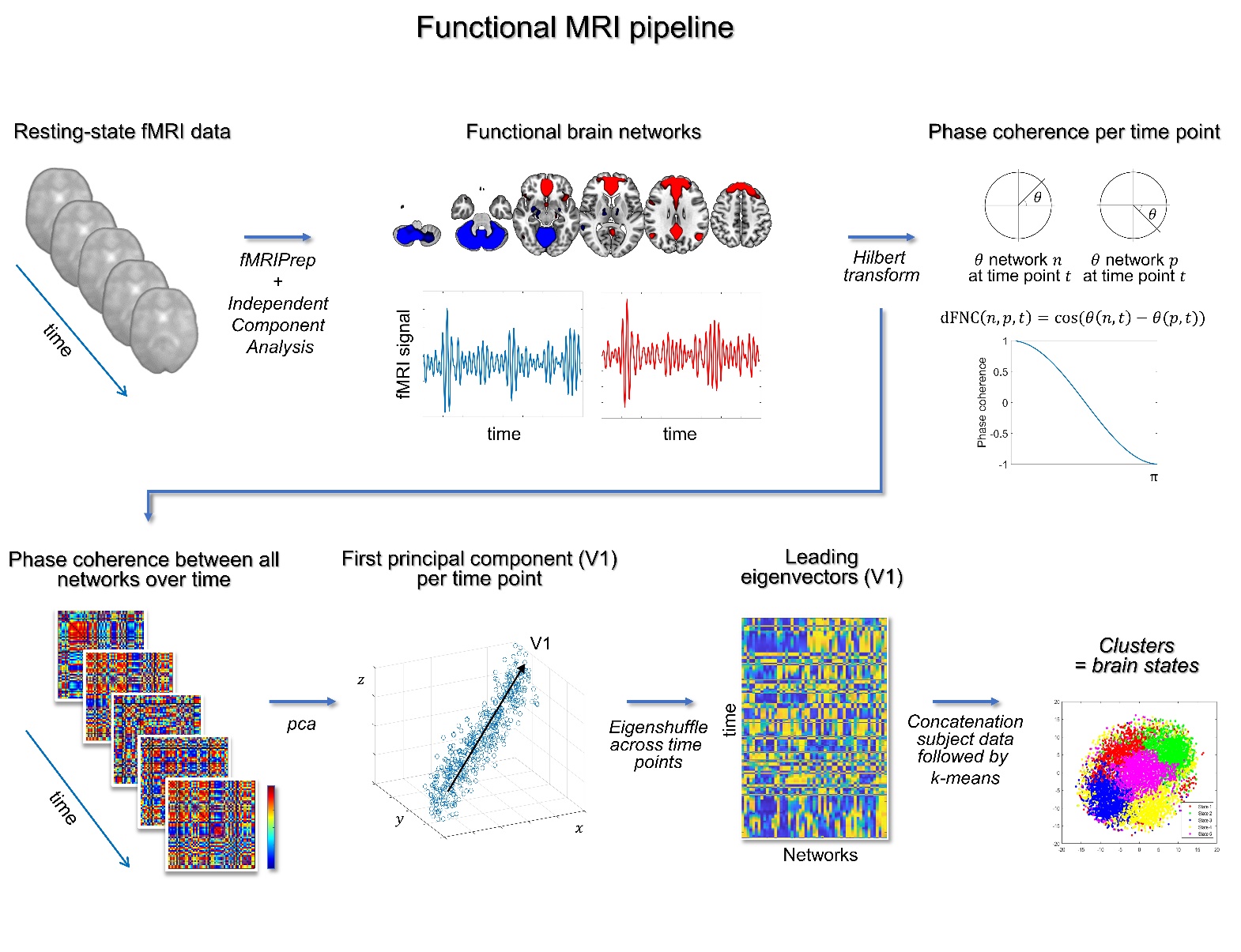

Supplement: S2 File — (DOCX) [file pone.0295984.s002.docx]

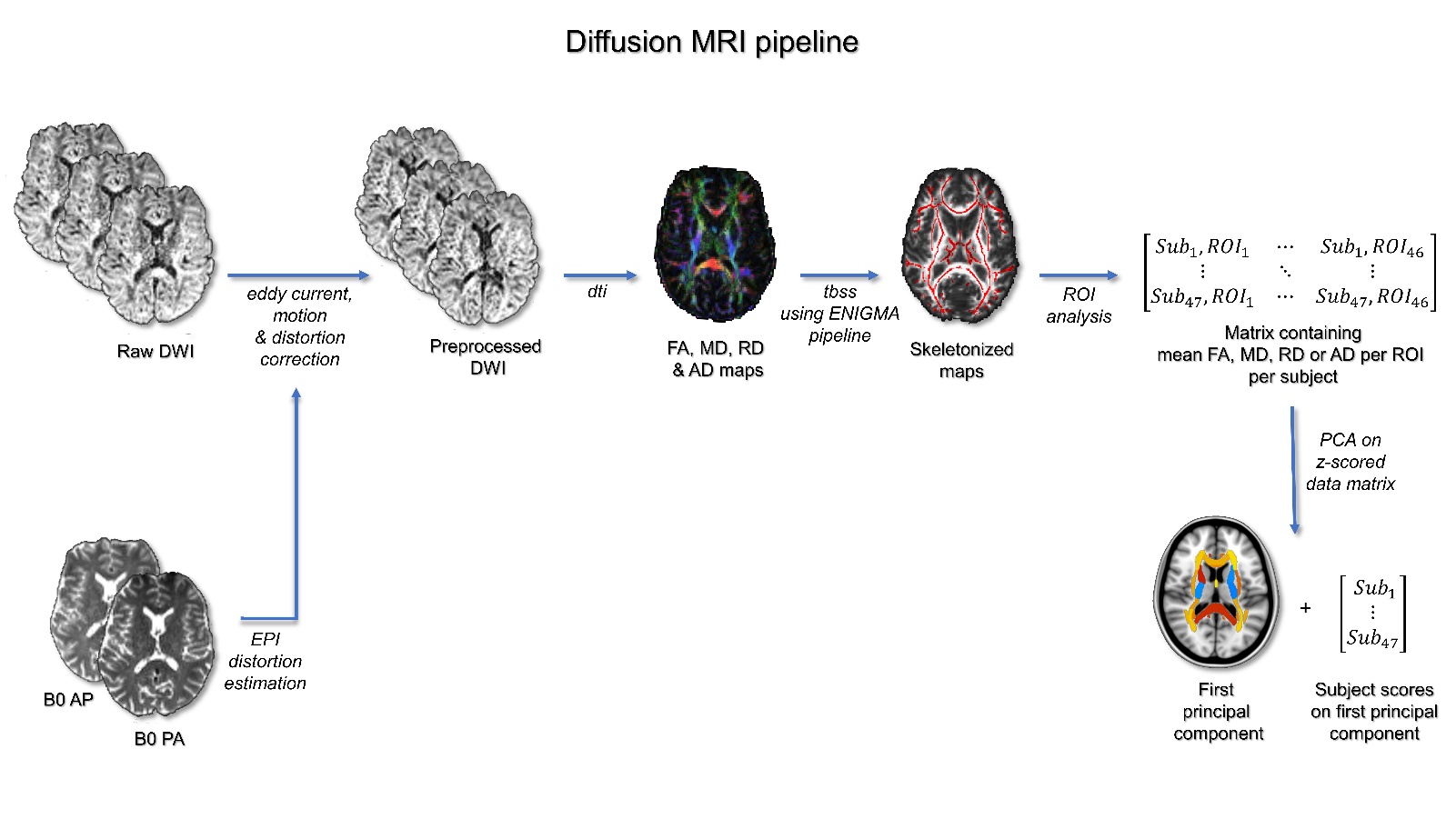

Supplement: S3 File — (DOCX) [file pone.0295984.s003.docx]

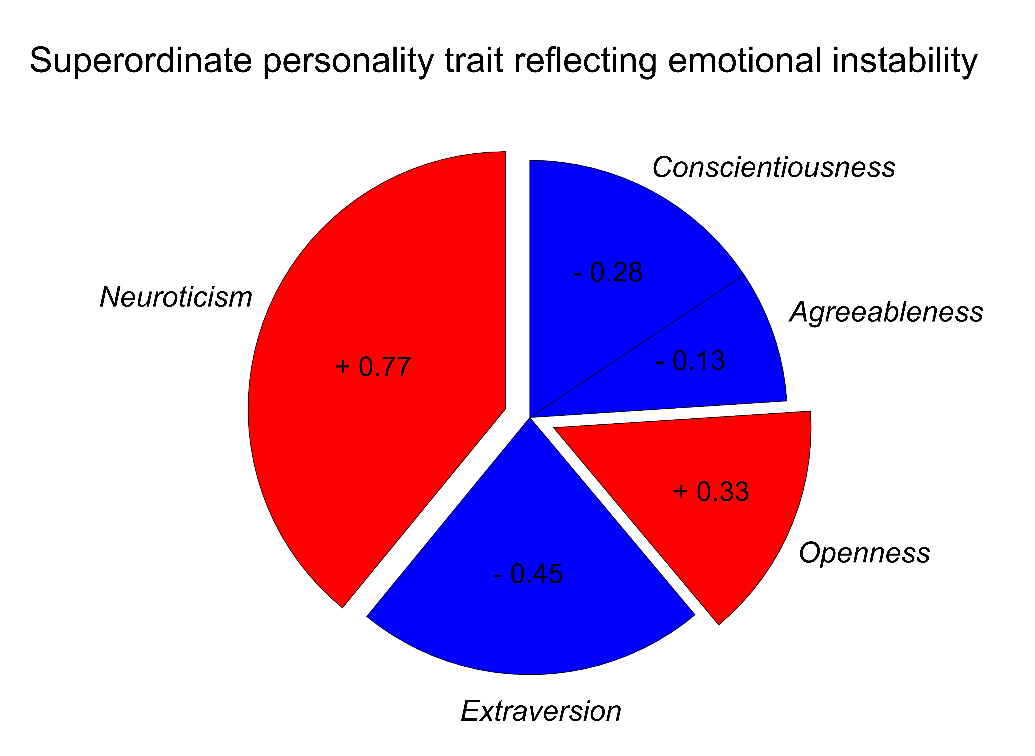


First principal component from Big Five personality data.

Supplement: S4 File — (DOCX) [file pone.0295984.s004.docx]
