# Supplementary material for: Dynamic phase-locking states and personality in sub-acute mild traumatic brain injury: An exploratory study
Source: PLoS One. 2023 Dec 15;18(12):e0295984. doi: 10.1371/journal.pone.0295984 (PMC10723684; doi:10.1371/journal.pone.0295984)
Supplement: S5 File — (DOCX) [file pone.0295984.s005.docx]

**Description of individual dynamic brain states**

The positive community in state 1 consists of visual and sensorimotor areas, while the negative community consists of frontal and subcortical areas.

State 2 seems to be the inverse of state 1, however, the frontal and subcortical regions (e.g., thalamus) are less pronounced in the positive community as compared to the negative community in state 1.

State 3 is characterized by a positive community consisting of insular, subcortical (basal ganglia), lateral prefrontal, supplementary motor, cingulate cortex, sensorimotor, and auditory areas, and a negative community consisting mainly of areas that are associated with the canonical default mode network (with medial prefrontal, posterior cingulate, and precuneus as main areas)

State 4 seems to be the inverse of state 3, however, there are noticeable differences, for example in the basal ganglia, which are less pronounced in the negative community as compared with the positive community of state 3.

State 5 contains elements of all aforementioned states, with the positive and negative community showing less segregation as compared to the other states.

We also computed the correlations between state centroids:

|  | *state 1* | *state 2* | *state 3* | *state 4* | *state 5* |
| --- | --- | --- | --- | --- | --- |
| *state 1* | 1.0000 -0.9513 0.1294 -0.1894 0.0446 | | | | |
| *state 2* | -0.9513 1.0000 -0.1613 0.0824 0.0088 | | | | |
| *state 3* | 0.1294 -0.1613 1.0000 -0.5326 -0.5054 | | | | |
| *state 4* | -0.1894 0.0824 -0.5326 1.0000 -0.3608 | | | | |
| *state 5* | 0.0446 0.0088 -0.5054 -0.3608 1.0000 | | | | |

These correlations show that state 1 and 2 are most alike (based on absolute correlation), as are state 3 to 5.
Thus, the states contain similar as well as different features (found in the same or opposite communities), and theoretically this seems plausible, since we use the entire brain at all times, with specific connections and networks changing in response to varying demands. Furthermore, the similarities between states may also be (partly) explained by the data parcellation technique used, namely ICA instead of for example ROI based techniques.
